# The Effect of Size on Ag Nanosphere Toxicity in Macrophage Cell Models and Lung Epithelial Cell Lines Is Dependent on Particle Dissolution

**DOI:** 10.3390/ijms15046815

**Published:** 2014-04-22

**Authors:** Raymond F. Hamilton, Sarah Buckingham, Andrij Holian

**Affiliations:** Department of Biomedical and Pharmaceutical Sciences, Center for Environmental Health Sciences, University of Montana, Missoula, MT 59812, USA; E-Mails: ray.hamilton@umontana.edu (R.F.H.J.); sarahgbuckingham@gmail.com (S.B.)

**Keywords:** silver nano, Ag, inflammasome, toxicity, macrophage, epithelial cell, particle uptake, dissolution

## Abstract

Silver (Ag) nanomaterials are increasingly used in a variety of commercial applications. This study examined the effect of size (20 and 110 nm) and surface stabilization (citrate and PVP coatings) on toxicity, particle uptake and NLRP3 inflammasome activation in a variety of macrophage and epithelial cell lines. The results indicated that smaller Ag (20 nm), regardless of coating, were more toxic in both cell types and most active in the THP-1 macrophages. TEM imaging demonstrated that 20 nm Ag nanospheres dissolved more rapidly than 110 nm Ag nanospheres in acidic phagolysosomes consistent with Ag ion mediated toxicity. In addition, there were some significant differences in epithelial cell line *in vitro* exposure models. The order of the epithelial cell lines’ sensitivity to Ag was LA4 > MLE12 > C10. The macrophage sensitivity to Ag toxicity was C57BL/6 AM > MARCO null AM, which indicated that the MARCO receptor was involved in uptake of the negatively charged Ag particles. These results support the idea that Ag nanosphere toxicity and NLRP3 inflammasome activation are determined by the rate of surface dissolution, which is based on relative surface area. This study highlights the importance of utilizing multiple models for *in vitro* studies to evaluate nanomaterials.

## 1. Introduction

Engineered nanomaterials (ENM) are valued for their unique physical characteristics. Of the ENM currently being used in consumer products a third of them are silver (Ag) nanomaterials [1]. The toxicity and bioactivity of internalized Ag nanomaterials are not well understood. Recent studies have demonstrated that there is a size dependency with smaller Ag nanomaterials being more toxic and more bioactive both *in vivo* and *in vitro* exposure models [1–4]. The rationale for this size-dependency varied depending on the study, and included increased reactive oxygen species (ROS) [3] and high relative surface area leading to increased Ag dissolution [1,4] associated with relatively smaller Ag nanomaterials.

Silver is well established as an antimicrobial agent as Ag ions are highly reactive, readily binding to sulfur and phosphate compounds [5]. Silver toxicity, however, is complex and generally divided into the effects of the Ag particles and Ag ions that are a byproduct of Ag particle oxidation and dissolution. Ag nanoparticles can interact with proteins to form stable silver/protein complexes, whereas Ag ions are generally not free and tend to form AgCl, Ag_2_S, or react with organic SH-groups [6]. The acidic pH of the biological environment can accelerate Ag dissolution such as in gastric acid [7] or lysosomal compartments [2]. In addition to the compounds stated above, Ag is known to react with reduced selenium species in biological models [7]. Silver nanomaterial exposure has been linked to amyloidosis in some animal exposure models [5], although the connection with human neurodegenerative disease has not been established.

Internalization of Ag into the cell is also divided into particle verses ionic form. Silver ions are reported to enter cells by copper (Cu) transporter proteins (Ctr1) [8], and possibly by the divalent metal transporter (DMT1) [6]. In contrast, Ag nanomaterials are internalized into cells by endocytosis [9], macropinocytosis [10], and/or passive diffusion [11]. Endocytosis is the most common mechanism for Ag nanoparticle internalization. Due to the overall negative zeta potential on Ag nanoparticles, scavenger receptors have been implicated as one possible mechanism associated with endocytosis, in addition to actin- and clathrin-dependent endocytosis [12]. Uptake of solid Ag nanoparticles typically results in the release of reactive Ag ions once the particles encounter the acidic pH in the lysosomal compartment of phagocytic cells. This apparently occurs more rapidly depending on the size of the Ag nanoparticles, with smaller particles dissolving faster inside the cell than larger particles, most likely due to increased surface area of the smaller particles [1]. This presents a unique toxicological problem as Ag nanoparticles are internalized as a solid that dissolves inside the cell potentially causing disruption of the phagolysosomal membrane resulting in inflammation and toxicity [2,13].

The cellular mechanism most associated with ENM-induced inflammation is activation of the NLRP3 inflammasome following phagolysosome rupture or compromise [14]. Rigid fibre-shaped ENM are most related to NLRP3 inflammasome activation [15–17], but some spherical or irregular shaped particles such as silica have also been implicated in this process [18]. There has been a report that Ag nanoparticles induced NLRP3 inflammasome activity in human monocytes [2]. Taken together, the NLRP3 inflammasome is an ideal marker for ENM bioactivity in cells, such as macrophages, that are capable of forming this complex.

This study used a set of four Ag nanoparticles/nanospheres (two sizes (20 and 110 nm) and two coatings (citrate and PVP)) to test the consistency of toxicity and particle uptake results obtained from various *in vitro* cell models, including two murine primary alveolar macrophages (C57Bl/6, and MARCO^−/−^), a transformed human monocyte-like cell line (THP-1), in addition to three murine lung epithelial cell lines (LA4, C10, and MLE12). The smaller Ag nanospheres were hypothesized to be more inflammatory/toxic regardless of coating due to the dissolution rate of the internalized particles. The absence of scavenger receptor MARCO was hypothesized to decrease particle uptake in macrophages in order to evaluate its role as a receptor for Ag nanoparticle endocytosis.

## 2. Results and Discussion

### 2.1. Toxicity and NLRP3 Inflammasome Activation in Macrophages Exposed to Ag Nanospheres

**Figure 1.**
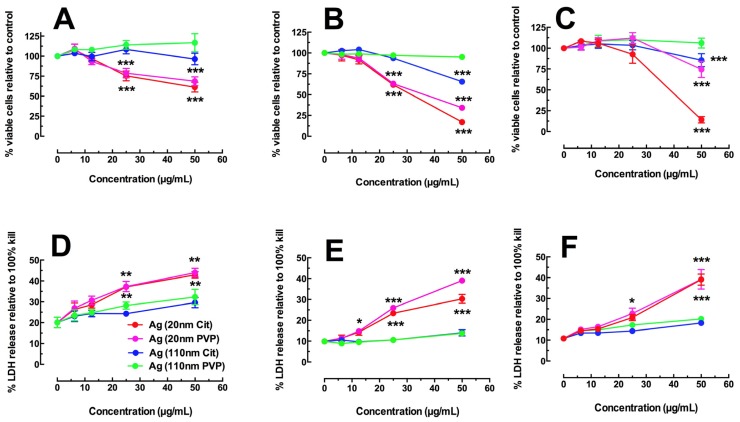
Toxicity of Ag nanospheres in three macrophage cell models using the MTS assay and LDH release assay at 24 h post particle exposure. (**A**) Percent viable cells compared to no particle (0 μg/mL) condition in the transformed THP-1 model; (**B**) Percent viable cells compared to no particle (0 μg/mL) condition in the C57BL/6 AM model; (**C**) Percent viable cells compared to no particle (0 μg/mL) condition in the MARCO null AM model; (**D**) Percent LDH release compared to 100% cell lysis in the transformed THP-1 model; (**E**) Percent LDH release compared to 100% cell lysis in the C57BL/6 AM model; (**F**) Percent LDH release compared to 100% cell lysis in the MARCO null AM model. All data expressed as mean ± SEM. Asterisks indicate significance at ^***^ *p* < 0.001, ^**^ *p* < 0.01, or ^*^ *p* < 0.05 compared to 0 μg/mL control condition by *post hoc* mean comparisons.

Three macrophage cell models were evaluated to study Ag toxicity and NLRP3 inflammasome activation *in vitro*. Transformed THP-1 cells are a human monocyte-like cell line that has been thoroughly characterized as a valid screening mechanism for the study of ENM by a number of laboratories [19]. Primary C57BL/6 AM and MARCO null AM on the C57 background were selected because they are commonly used primary cell models that would also allow for the determination of scavenger receptor involvement in Ag particle uptake similar to crystalline silica uptake that has been characterized elsewhere [20]. Figure 1 shows the toxicity data for all three AM models using two assays (MTS assay and LDH release). The Ag nanosphere dose range was between 0 and 50 μg/mL (0, 6.25, 12.5, 25, and 50 μg/mL) for 24-h exposures as described in the Experimental Section.

Regardless of the cell model and metric used, 20 nm Ag were significantly toxic at the higher dose ranges (Figure 1A–F). The coating did not have any significant effects on particle toxicity, however the citrate stabilized materials tended to be more toxic than the PVP. The order of toxicity was consistent in all cell models: 20 nm citrate ≥ 20 nm PVP > 110 nm citrate ≥ 110 nm PVP. The 110 nm Ag nanoparticles were essentially non-toxic in this dose range. MARCO null AM was the least sensitive model, as the significant toxicity by 20 nm Ag nanospheres was only apparent at the highest dose tested (Figure 1C). In contrast, the significant 20 nm Ag toxicity started at 25 μg/mL for THP-1 and C57BL/6 AM cells (Figure 1A,B respectively). The LDH results in Figure 1D–F were consistent with the MTS assay, but the resolution and detail was poor with this assay possibly due to a potential degradation of the LDH by Ag.

**Figure 2.**
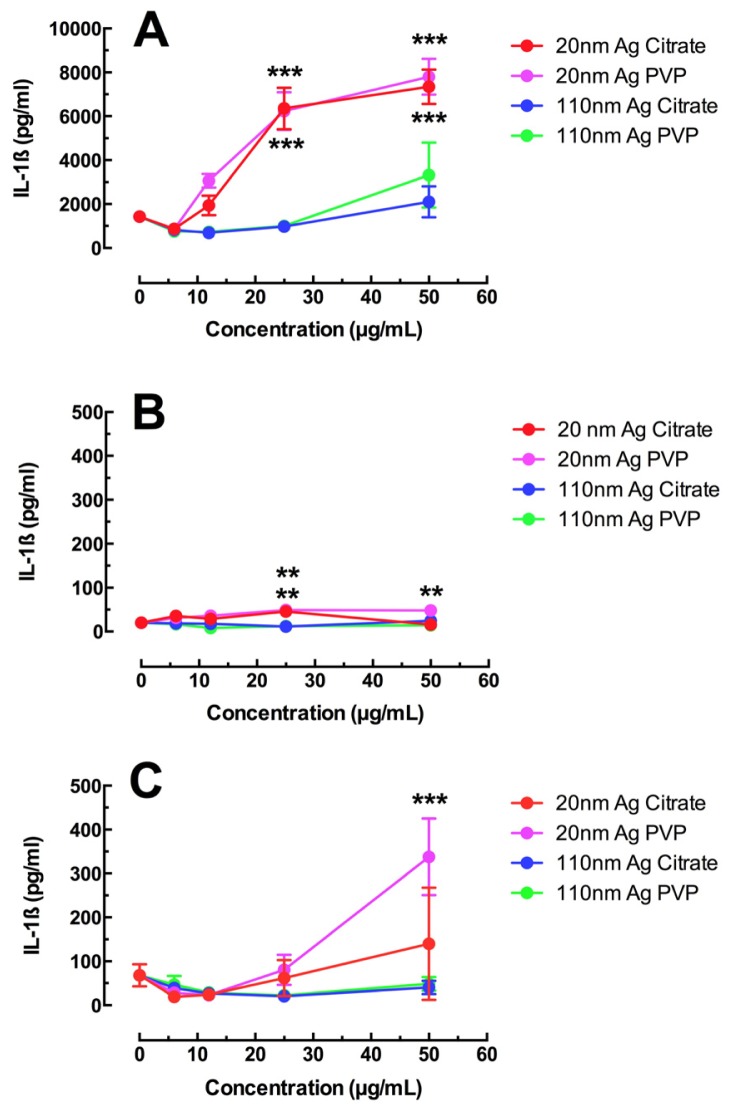
NLRP3 activation in three macrophage cell models using IL-1β release as a proxy measure. (**A**) IL-1β release from transformed THP-1 cells 24 h post Ag exposure; (**B**) IL-1β release from C57BL/6 AM 24 h post Ag exposure; (**C**) IL-1β release from MARCO null AM 24 h post Ag exposure. All data expressed as mean ± SEM. Asterisks indicate significance at ^***^ *p* < 0.001, or ^**^ *p* < 0.01, compared to 0 μg/mL control condition by *post hoc* mean comparisons.

NLRP3 inflammasome activation (measured as IL-1β release from macrophages) was tested in all three AM exposure models and the results are shown in Figure 2. THP-1 cells have been established as a very sensitive indicator of ENM inflammatory potential [17,21,22]. NLRP3 inflammasome results in this *in vitro* exposure cell model were consistent with the toxicity data indicating a dose-response increase in IL-1β release from THP-1 cells for the two 20 nm Ag materials at the highest doses tested (25 and 50 μg/mL) as shown in Figure 2A. The highest concentration of 110 nm Ag showed increases in IL-1β, but it was not statistically significant. IL-1β release from C57BL/6 AM was subdued in comparison to the response of THP-1 cells, although the results with 20 nm Ag did reach statistical significance at higher particle concentrations (Figure 2B). This result was not necessarily typical for murine AM isolated from all strains of mice, since isolated Balb/c AM responded similarly to THP-1 cells (data not shown). The MARCO null AM result was highly variable, but roughly similar to the C57BL/6 AM result with a significant increase for the 20 nm Ag at the highest concentration (Figure 2C). The 110 nm Ag nanoparticles regardless of coating had no effect on NLRP3 inflammasome activation in either primary macrophage cell model. Taken together, the toxicity and inflammasome assessment for these cell models indicated that 20 nm Ag was more toxic and more bioactive than 110 nm Ag materials regardless of coating. This is entirely consistent with other studies that implied that smaller Ag nanoparticles were more toxic [1,3,4,13]. In addition, the size-dependent NLRP3 inflammasome activation shown in human monocytes [2], supports the observations in this study, especially in the THP-1 cell model. Clearly, the smaller Ag nanoparticles were more bioactive.

The relative volume and surface areas of these Ag nanospheres can affect the results since the comparisons were based solely on mass. For any given mass there will be 166 more of the 20 nm Ag nanospheres compared to the 110 nm Ag materials when corrected for volume as described [1]. However, when correcting for surface area, the relative surface area for 20 nm Ag nanospheres at any given mass is 5.48 times that of 110 nm Ag nanospheres. That increase in relative surface area alone could account for the elevated IL-1β results seen in Figure 2A as the ED50 for 20 nm Ag regardless of coating, was roughly 6 times that of 110 nm Ag nanospheres at the same dose.

### 2.2. Uptake of Ag Nanospheres by Murine Macrophages

**Figure 3.**
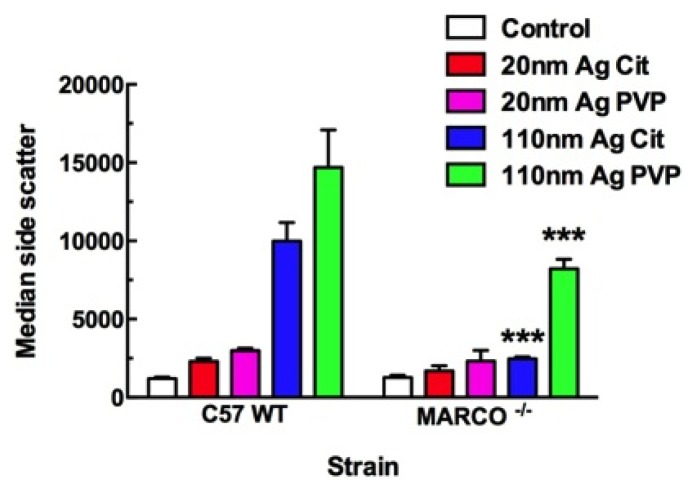
Ag uptake at 1 h in murine AM cells using side scatter measured by flow cytometry. C57BL/6 and MARCO null AM were exposed to Ag particles in suspension culture for 1 h. All data expressed as mean ± SEM. Asterisks indicate significance at ^***^ *p* < 0.001 compared corresponding particle exposure condition by *post hoc* mean comparisons.

Primary macrophage cell models were used to study particle uptake, because THP-1 cells are extremely adherent and difficult to maintain in the suspension cultures necessary to quantitate internalization. Spherical particle uptake in macrophage cells can be monitored with light side scatter on a flow cytometer as previously described [20]. Larger particles naturally produced more light scatter; however, uptake of both Ag sizes can be assayed with this technique. Figure 3 shows the median light side scatter for AM cells exposed to Ag nanospheres (25 μg/mL) for 1 h in a suspension culture as described in the Experimental Section. There was a clear decrease in particle uptake by MARCO null AM cells, which reached statistical significance for the larger 110 nm nanospheres regardless of coating. The citrate Ag 110 nm nanoparticles were essentially excluded from MARCO null AM, and PVP Ag 110 nm nanoparticle uptake was significantly reduced in these cells. The uptake of 20 nm Ag nanospheres were reduced up to 50% in MARCO null AM, but not to a significant degree.

Table 1 shows the hydrodynamic diameter of the various forms of the Ag nanospheres in media with variable amounts of serum, as determined by DLS. Interestingly, 110 nm Ag nanoshperes, regardless of coating, did not aggregate to any significant degree. The 20 nm Ag nanospheres aggregated slightly, such that the 20 nm aggregate diameters were virtually indistinguishable from the 110 nm non-aggregated nanoparticles. This would suggest that the uptake mechanism for both sizes was most likely endocytosis, since the net particle size in the cell cultures were similar.

**Figure 4.**
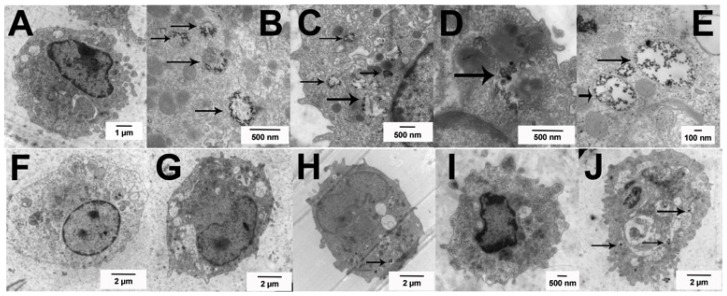
Uptake of Ag nanospheres by C57BL/6 and MARCO null AM illustrated using TEM imaging at 1.5 h post exposure. (**A**) No particle control C57BL/6 AM; (**B**) C57BL/6 AM exposed to 25 μg/mL 20 nm citrate-stabilized Ag nanospheres; (**C**) C57BL/6 AM exposed to 25 μg/mL 20 nm PVP-stabilized Ag nanospheres; (**D**) C57BL/6 AM exposed to 25 μg/mL 110 nm citrate-stabilized Ag nanospheres; (**E**) C57BL/6 AM exposed to 25 μg/mL 110 nm PVP-stabilized Ag nanospheres; (**F**) No particle control MARCO null AM; (**G**) MARCO null AM exposed to 25 μg/mL 20 nm citrate-stabilized Ag nanospheres; (**H**) MARCO null AM exposed to 25 μg/mL 20 nm PVP-stabilized Ag nanospheres; (**I**) MARCO null AM exposed to 25 μg/mL 110 nm citrate-stabilized Ag nanospheres; (**J**) MARCO null AM exposed to 25 μg/mL 110 nm PVP-stabilized Ag nanospheres. Arrows indicate areas of organized Ag particle uptake with the cells.

The TEM images in Figure 4 completely support this observation. Primary AM were cultured for 1.5 h in suspension culture ± Ag at 25 μg/mL, prior to fixation and processing for TEM imaging as described in the Experimental Section. The C57BL/6 AM took up Ag nanoparticles regardless of size and/or coating in organized vesicles with the appearance of clustered particles obvious throughout the cell (high magnification inside cells—Figure 4B–E). Based on sizing data, the 20 nm Ag nanospheres appear to be already dissolved down to the 7 nm gold core by this time (1.5 h). In contrast, the larger 110 nm Ag nanospheres were starting to dissolve as was apparent in Figure 4D for the 110 nm citrate Ag nanospheres. MARCO null AM are shown as wide-angle whole cell images to demonstrate the relative absence of particles throughout the cell. The citrate Ag nanoparticles regardless of size were not readily detectible within cells (Figure 4G,I). PVP Ag nanoparticles were only sparsely detectible within the cell. This imaging was consistent with the side scatter data in that the MARCO null AM showed reduced particle uptake. This suggests that the Class IA scavenger receptor, in this case MARCO, was involved with the endocytosis of Ag nanoparticles probably due to the negative surface charge. This result has been demonstrated in another study with J774A macrophages [12]. The citrate-coated particles are more negatively charged and that probably accounted for the subtle difference in the results with the MARCO null AM particle uptake (see zeta potential, Table 2). With the results taken together, the decreased uptake of Ag by MARCO null AM correlates with decreased toxicity in MARCO null AM at all doses except the highest dose of 20 nm Ag citrate nanoparticle. The results at the highest doses are more difficult to interpret. Apparently, some Ag particle uptake occurs by an alternative mechanism(s) similar to what has been reported for silica uptake in the absence of MARCO [20]. While uptake through MARCO appears to be important to the final outcomes, uptake by alternative mechanisms could also be important pathways to increased phagolysosomal permeability.

**Figure 5.**
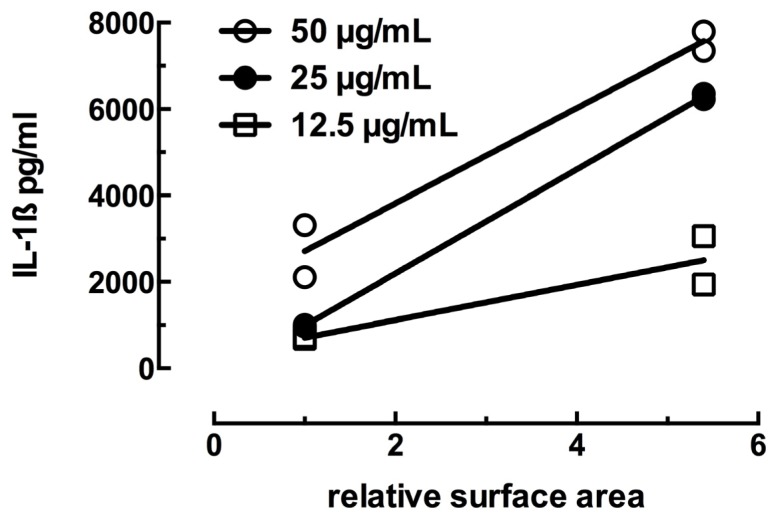
Correlation plot of Ag nanoparticle relative surface area (1 = 110 nm and 5.48 = 20 nm), and IL-1β production by THP-1 cells. Significant regression plots at two highest concentrations.

The C57BL/6 AM high magnification images of dissolved and dissolving Ag nanospheres in Figure 4B–E indicate that the particle dissolution rate is consistent with, and possibly critical to, the toxicity and inflammasome activation potential of the Ag nanospheres. This finding is consistent with the particle dissolution hypothesis proposed by several researchers [1,4]. It does not exclude the involvement of ROS in the process [3], but it may suggest that ROS is an “effect of toxicity” and not a direct cause of the toxicity. The smaller Ag nanospheres dissolve rapidly when the pH becomes acidic upon phagolysosome fusion. This quick release of reactive Ag ions compromises the phagolysosome resulting in cathepsin B release and NLRP3 inflammasome activation as described above and in human monocytes [2]. The larger Ag nanospheres dissolve more slowly in the lysosome environment due to the reduced exposed surface area of the larger particles for the same mass resulting in a lower delivery of Ag ions. The rate of Ag nanosphere dissolution would be directly proportional to the available surface area per unit mass, which is much higher (5.48×) in the small Ag nanospheres. This becomes more evident if the relative surface area is correlated to the IL-1β production in the most sensitive cell model, THP-1. Figure 5 shows plots of the mean IL-1β production plotted against the relative surface area with 1 representing both 110 nm Ag particles and 5.48 representing the relative surface area of the 20 nm Ag particles. The resulting regression plots indicated significant correlations in the two highest concentrations (25 and 50 μg/mL) with *r*_2_ of 0.99 and 0.97 respectively. The two lower concentrations did not achieve statistical significance (6.25 μg/mL data not shown).

### 2.3. Toxicity of Ag Nanospheres in Murine Lung Epithelial Cell Lines

**Figure 6.**
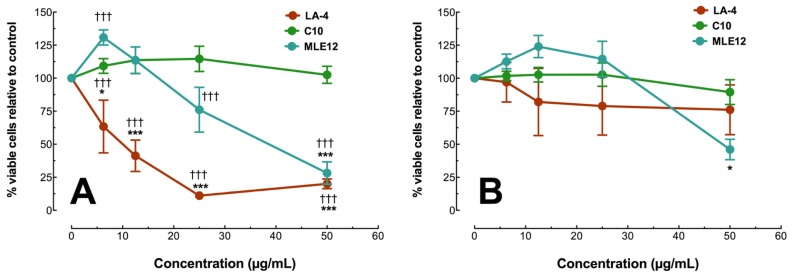
Toxicity of citrate-stabilized Ag nanospheres in three murine epithelial cell models using the MTS assay 24 h post particle exposure. (**A**) Percent viable cells with 100% confluent murine epithelial Type II cells exposed to 20 nm Ag nanospheres; (**B**) Percent viable cells with 100% confluent murine epithelial Type II cells exposed to 110 nm Ag nanospheres. All data expressed as mean ± SEM % viable cells relative to no particle (0 μg/mL) condition. Asterisks indicate significance at ^***^ *p* < 0.001, or ^*^ *p* < 0.05 compared to 0 μg/mL control condition by *post hoc* mean comparisons. Daggers indicate significance at ††† *p* < 0.001 compared to the other cell lines at a specific concentration.

Due to the lack of significant difference between the Ag nanosphere stabilizers on macrophage toxicity, epithelial cell exposures were only conducted with the citrate-stabilized forms of the Ag nanospheres. Epithelial cells were seeded a day before Ag nanosphere exposure and the seeding densities were optimized to generate a 100% confluent cell culture as described in the Experimental Section. Figure 6 shows cell viability for three murine lung epithelial cell lines exposed to citrate-stabilized Ag nanospheres for 24 h as assessed by MTS assay (LDH results were too variable to be useful). Particle concentrations were identical to the macrophage study with 50 μg/mL the maximal dose. There were significant differences in toxicity to 20 nm Ag (Figure 6A) between cell lines. The order of sensitivity to 20 nm Ag was LA4 > MLE12 > C10. There was complete absence of toxicity in C10 cells. In contrast, with the 110 nm Ag nanosphere exposures, none of the cell lines, with the exception of the MLE12 at the highest dose, showed significant toxicity (Figure 6B). In general, the 20 nm Ag nanospheres were again more toxic with the one exception of the resistant C10 cell line (Figure 6A).

### 2.4. Uptake of Ag Nanospheres in Murine Lung Epithelial Cell Lines

**Figure 7.**
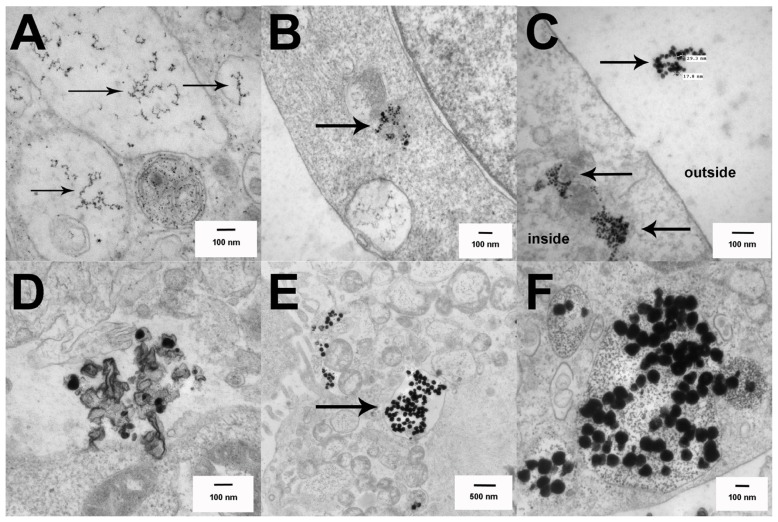
Uptake of citrate-stabilized Ag nanospheres in murine epithelial cell lines demonstrated by TEM imaging at 4 h post exposure. (**A**) LA4 cells exposed to 20 nm Ag nanospheres; (**B**) C10 cells exposed to 20 nm Ag nanospheres; (**C**) MLE12 cells exposed to 20 nm Ag nanospheres; (**D**) LA4 cells exposed to 110 nm Ag nanospheres; (**E**) C10 cells exposed to 110 nm Ag nanospheres; (**F**) MLE12 cells exposed to 110 nm Ag nanospheres. Arrows indicate areas of clustered Ag particles.

In order to determine if Ag dissolution was similar in the epithelial cells compared to the macrophages, TEM imaging was conducted on the three cell lines at 4 h post Ag exposure (25 μg/mL). Figure 7 shows the nanoparticles inside cells on high magnification TEM. Similar to the AM data, Ag nannoparticles, regardless of size, were taken up in organized fashion with nanoparticles clustered in internalized vesicles (e.g., phagolysosomes). The smaller 20 nm Ag had already dissolved down to the 7 nm gold cores at this time (Figure 7A–C). The cell line with the most toxicity (LA4) showed larger vacuoles with the particles starting to separate and distribute throughout the cell (Figure 7A) possibly due to phagolysosome breakdown. In contrast, the 20 nm Ag particles in the other two cell lines (C10 and MLE12) appeared tightly clustered but clearly dissolved down to the gold cores (Figure 7B,C). Figure 7C shows the inside and outside of a MLE12 cell with 20 nm Ag nanospheres present on both sections. This plainly illustrates that the external to the cell particles were intact at roughly 20 nm, whereas the internalized particles were closer to the 7 nm gold core dimensions, demonstrating that Ag nanoparticle dissolution occurred inside the cell. Figure 7D shows 110 nm Ag particles in the process of breaking down inside a LA4 cell. Figure 7E,F show 110 nm Ag nanospheres intact and collected in organized structure within C10 and MLE12 cells, respectively.

**Figure 8.**
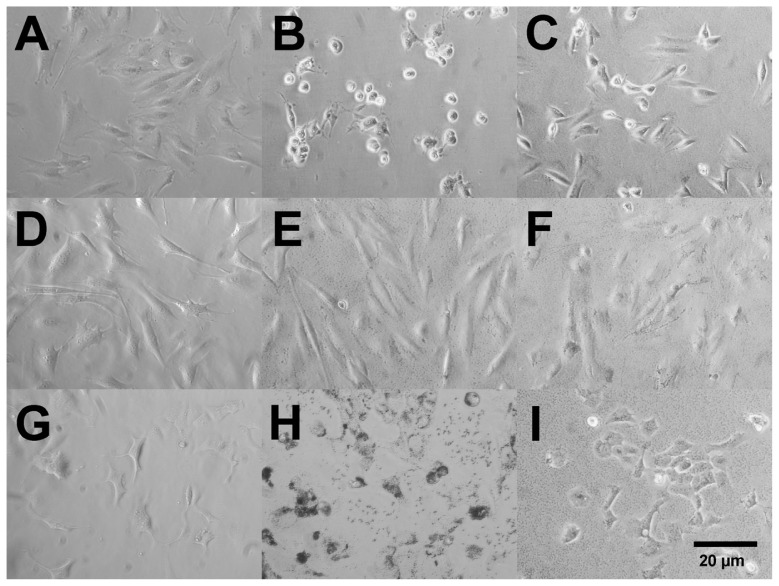
Phase contrast images of confluent murine epithelial cells at 4 h post particle exposure. (**A**) Control LA4 cells with no particle exposure; (**B**) LA4 cells exposed to 50 μg/mL 20 nm citrate-stabilized Ag nanospheres; (**C**) LA4 cells exposed to 50 μg/mL 110 nm citrate-stabilized Ag nanospheres; (**D**) Control C10 cells with no particle exposure; (**E**) C10 cells exposed to 50 μg/mL 20 nm citrate-stabilized Ag nanospheres; (**F**) C10 cells exposed to 50 μg/mL 110 nm citrate-stabilized Ag nanospheres; (**G**) Control MLE12 cells with no particle exposure; **(H**) MLE12 cells exposed to 50 μg/mL 20 nm citrate-stabilized Ag nanospheres; (**I**) MLE12 cells exposed to 50 μg/mL 110 nm citrate-stabilized Ag nanospheres. Magnification at 200× for all images.

Figure 8 shows phase contrast images of the three cell lines with and without the two Ag nanospheres 4 h post particle exposures. The early cytotoxicity caused by Ag nanoparticles in LA4 cells was evident as the cells were starting to “round up” with a white outline in the phase contrast images (Figure 8B,C). The C10 cells, in contrast, showed no visible effect from the internalized Ag nanospheres (Figure 8E,F), regardless of size. The MLE12 cells showed aggressive uptake of the Ag material, but no indication of toxicity at this time (Figure 8I,J). The order of sensitivity to the smaller Ag nanospheres was LA4 > MLE12 > C10. The amount of serum in these cultures varied, and this has been cited as a factor in Ag dissolution [1], although the time frame for significant serum-dependent solubilization is several hours later than the time points illustrated here. The serum-initiated Ag dissolution would most likely result in detoxification of the particles causing Ag ion formation outside of the cell with likely binding to serum proteins preventing entry into cells. The relative serum concentrations in culture media were: LA4 > C10 > MLE12 and would therefore not appear to account for the order of epithelial cell sensitivity. The relative amount of Ag nanospheres taken up by the epithelial cell lines also differed: MLE12 > C10 ≈ LA4 (Figure 8). Taken together, the toxicity of 20 nm Ag cannot be attributed to the amount of particles taken in or the amount of serum in the culture media, as there is no association between those factors and toxicity. Instead, the toxicity of 20 nm Ag nanospheres was directly related to the rate of dissolution in the acidic lysosomes, which varied from one cell type to the next. The more rapid dissolution of Ag in LA4 cells was apparent in Figure 7D as the 110 nm Ag nanospheres appear to be dissolving faster than in the other two murine lung epithelial cell lines. Taken together, the results would suggest that the sensitivity of the different epithelial cells lines was more likely due to differences in phagolysosomal acidification rates and/or differences in Ag ion toxicity among the cell lines, which could be related to the differential sources, lung areas, and conditions that the cells were originally obtained from.

## 3. Experimental Section

### 3.1. Silver Nanoparticle Characterization

Ag nanoparticles obtained from NCI Nanotechnology Characterization Laboratory (NCL, Frederick, MD, USA) were originally supplied from nanoComposix, Inc. from the BioPure™ (San Diego, CA USA) line of materials. Nanoparticle size was characterized by the NCL. Detailed characterization can be found in Table 2. The four Ag nanoparticles include: nominal 20 nm diameter, citrate stabilized colloidal Ag; nominal 20 nm, polyvinlypyrrolidone (PVP) stabilized colloidal Ag; nominal 110 nm, citrate stabilized colloidal Ag; and Nominal 110 nm, PVP stabilized colloidal Ag. The citrate-stabilized particles were supplied in 2 mM citrate buffer at a concentration of 1 mg/mL. The PVP-stabilized particles were supplied in water at a concentration of 1 mg/mL. All particles were manufactured on a 7 nm gold (Au) core by a proprietary process.

### 3.2. Cell Isolation and Culture

All cell cultures were maintained in a 37 °C water-jacketed incubator with 5% CO_2_ (ThermoForma, Houston, TX, USA), unless otherwise noted. Three murine epithelial cell lines were maintained in various media with varying serum concentrations. The C10 (C1C10) cell line is a non-tumorigenic clone of the NAL-1A type II pnuemocyte cell line derived from the alveolar region of a normal Balb/c mouse lung. The C10 cell line was maintained in RPMI + 10% fetal bovine serum (FBS) with A/A (Antibiotics/Antimycotic: 10,000 IU/mL penicillin G, 10 mg/mL streptomycin sulfate, and 25 μg/mL amphotericin B from MediaTech, Manassas, VA, USA). The MLE12 cell line is a SV40 transformed lung epithelial cell line from the distal airways of the FVB/N mouse. The MLE12 cell line was maintained in HITES medium (50:50 mixture of DMEM and Ham’s F12 with 0.005 mg/mL insulin, 0.01 mg/mL transferrin, 30 nM sodium selenite, 10 nM hydrocortisone, 10 nM β-estradiol, 10 mM HEPES, and additional 2 mM l-glutamine) + 2% FBS and A/A. The LA-4 lung epithelial cell line from an A/He mouse adenoma was maintained in Ham’s F-12K (Kaighn’s) + 15% FBS with A/A.

The THP-1 acute monocytic leukemia cell line from human peripheral blood was maintained in RPMI + 10% FBS with A/A. Prior to particle exposure experiments, the THP-1 cells in suspension were differentiated into a macrophage-like cell by adding 150 nM 1,25-dihydroxyvitamin D_3_ (EMD Millipore, Darmstadt, Germany) for 24 h. The resulting semi-adherent cells were harvested in the existing media with a cell scraper and centrifuged at 400× *g* for 5 min. The resulting cell pellet was re-suspended in 1 mL of complete media, and a 40 μL sample was then counted on a Z2 Coulter Counter (Beckman Coulter, Miami, FL, USA). The cells were suspended at 1 × 10^6^ cells/mL and a small amount of phorbol 12-myristate 13-acetate (5 nM PMA, Sigma, St. Louis, MO, USA) and lipopolysaccharide (10 ng/mL LPS, Sigma, St. Louis, MO, USA) was added to stimulate aggressive phagocytosis and NFκB activation respectively. Full characterization of this model for ENM toxicity and NLRP3 inflammasome activation can be found elsewhere [19].

C57Bl/6 and macrophage receptor with collagenous structure (MARCO)-null on C57Bl/6 background mice (2-months old, male) were housed in controlled environmental conditions (22 ± 2 °C; 30%–40% humidity, 12-h light: 12-h dark cycle) and provided food and water *ad libitum*. All procedures were performed under protocols approved by the IACUC of the University of Montana. Mice were euthanized by sodium pentobarbital (Euthasol™, Virbac Corp., Fort Worth, TX, USA), and the lungs with the heart were removed. Lung lavage was performed using ice-cold PBS (pH 7.4). Lung lavage cells were isolated by centrifugation (400× *g*, 5 min, 4 °C) and cell counts obtained using a Coulter Z2 particle counter (Beckman Coulter, Miami, FL, USA). The alveolar macrophages (AM) cells were suspended in RPMI + 10% FBS with A/A at 10^6^ cells/mL. 20 ng/mL LPS was added to stimulate pro-IL-1β formation (via NFκB translocation to AM nucleus).

### 3.3. Silver Nanoparticle Exposure

Ag nanoparticles were sonicated for ten min in supplied media using a jewelry-cleaner type sonicator before cell treatments to ensure thorough dispersion. All cells were treated with 0, 6.25, 12.5, 25, or 50 μg/mL of Ag nanoparticles. Epithelial cells seeded on 96-well tissue culture plates at densities of 15,000 cells/well reached approximately 100% cell confluency in 24 h, when they were exposed to Ag nanoparticles for an additional 24 h. Vitamin D_3_-transformed, PMA/LPS-treated THP-1 cells or LPS-treated primary AM were exposed to Ag nanoparticles in 1.5 mL microcentrifuge tubes. The resulting suspension was mixed by pipette action, transferred to 96-well plates at 100 μL/well (1 × 10^5^ cells/well), and incubated for 24 h. The resulting supernatants of all macrophage cell types was collected and stored for IL-1β ELISA.

### 3.4. Cytotoxicity

Cell viability was determined by MTS reagent using the CellTiter96 assay (Promega, Madison, WI, USA) according to the manufacturer’s protocol with the below exception. In order to avoid artifacts in the optical density (OD) values, steps were taken to remove the MTS reagent (transferring it into another plate) to remove obstruction of cell/particle material during absorbance measurements at 490 nm. All Ag particle exposed cells were compared to the no particle control condition and expressed as percent viable cells relative to control condition. It is possible to have greater than 100% viability, which indicates cell proliferation. Cell membrane permeabilization in the macrophage models only was determined by release of lactate dehydrogenase (LDH) using the CytoTox96 assay (Promega, Madison, WI, USA) according to manufacturer’s protocol. Percent LDH release was expressed relative to 100% total lysed cells (cell lysis done 1 h before assay with provided reagent) at the same cell concentration.

### 3.5. IL-1β Release

Mouse and human IL-1β DuoSets were obtained from R&D Systems (Minneapolis, MN, USA) and ELISA assays were performed according to the manufacturer’s protocol. Absorbance was measured at 450 nm and data expressed as pg/mL of culture supernatant.

### 3.6. Dynamic Light Scattering (DLS) to Assess Ag Aggregates in Media

Particle size was measured at 25 °C using a Malvern Zetasizer nano ZS90 (Malvern Instruments, Worcestershire, UK) at a 90° detector angle. Samples consisted of 1 mL of RPMI culture media with variable serum concentrations and 25 μg/mL Ag nanoparticles. Nanoparticles were sonicated for 10 min prior to addition. All samples vortexed before each reading.

### 3.7. Silver Nanoparticle Uptake

Primary murine alveolar macrophages (AM) were exposed to 25 μg/mL of Ag nanoparticles and placed on a rotator at 37 °C for 1 h. Cells were washed with PBS to remove excess extracellular particles. Ag nanoparticle uptake was assessed by side scatter angle (SSC) using flow cytometry (FACSAria™, BD, Franklin Lakes, NJ, USA) as described elsewhere [20]. This technique does not distinguish internalized particles from particles stuck on the cell surface. However, TEM done at approximately the same time did not indicate a large number of Ag nanoparticles stuck to the surface. Therefore, it was assumed that the sidescatter values reflected internalized nanoparticles.

Epithelial cells were seeded in 6-well plates at 250,000 cells/well and incubated for 24 h. The Ag nanoparticles were added at 50 μg/mL, and cells were incubated for an additional 4 h. Cells were scraped and fixed in 2.5% EM grade glutaraldehyde in cacodylate buffer at pH 7.2. The cells were rinsed in dH2O and resuspended in 1% osmium tetroxide for 1 h and rinsed in dH2O. The cells were dried in a graded ethanol series followed by embedding of the cell pellet in epoxy. Thin sections were stained with 2% uranyl acetate for 30 min at room temperature, rinsed in dH2O, and stained for 5 min with Reynolds lead citrate stain. The cells were imaged in a Hitachi H-7100 transmission electron microscope at 75 kV. For AM TEM, isolated AM were exposed to 25 μg/mL Ag nanoparticles for 1.5 h in a suspension culture as described above. They were centrifuged in a microfuge, washed once in PBS and processed as described for the epithelial cell TEM.

### 3.8. Statistical Analyses

Statistical analyses involved comparison of means using a one or two-way ANOVA followed by Tukey’s test to compensate for increased type I error. Correlations and linear regressions were performed for mean THP-1 IL-1β production and the Ag relative surface area at different particle concentrations. All probabilities were two-tailed unless otherwise stated. Statistical power was greater than 0.8. Statistical significance was defined as a probability of type I error occurring at less than 5% (*p* < 0.05). The minimum number of experimental replications was 3–4 depending on the experiment.

## 4. Conclusions

Overall, our study agrees with recent publications supporting the notion that smaller Ag nanomaterials are more toxic and bioactive regardless of the cell model used. Our TEM imaging results suggest that Ag nanospheres, once internalized, were dissolving in the relatively low pH within the acidic phagolysosomal environment in the cell. Smaller Ag nanospheres appear to dissolve more rapidly and more completely resulting in enhanced toxicity, most likely due to the increased rate of Ag ions released within the phagolysosomal compartment compared to the slower rate of Ag dissolution rate of the larger Ag particles since a lower surface area was expressed. There was no significant effect of Ag stabilization or coating with Ag size being the more important variable. The MARCO null AM results (decreased particle uptake and reduced toxicity) indicated that class A scavenger receptors were involved in recognizing Ag nanomaterials and internalizing the particles by endocytosis in cells that expressed this receptor. Finally, this study’s results imply that there was a high degree of variability from one cell model to the next, and care must be exercised when choosing and interpreting the results from any one model. This is not unique to Ag as this has been reported with other ENM in similar cell models [23]. If possible, more than one cell model should be used to fully characterize any ENM biological impact. It should also be noted that these Ag materials were formed about a gold core that could affect the overall stability of the Ag nanomaterials.

## Figures and Tables

**Table 1.** Aggregation state of Ag nanospheres in RPMI culture medium with variable serum concentrations by DLS. Data are mean hydrodynamic diameter (nm) ± range (nm).

Sample	2% Serum	10% Serum	15% Serum
Media background no Ag *	31.1 ± 24 nm	30.9 ± 25.5 nm	46.8 ± 23.9 nm
Media + 20 nm citrate Ag	129.8 ± 78.0 nm	131.6 ± 63.0 nm	89.7 ± 47.5 nm
Media + 20 nm PVP Ag	124.0 ± 95.0 nm	136.6 ± 87.8 nm	97.1 ± 62.0 nm
Media + 110 nm citrate Ag	165.2 ± 44.2 nm	164.2 ± 44.8 nm	161.6 ± 44.5 nm
Media + 110 nm PVP Ag	168.1 ± 42.9 nm	171.9 ± 43.6 nm	173.5 ± 48.5 nm

* data values reflect very low intensity peaks, which may be the noise floor and not indicative of actual particulate matter.

**Table 2.** Physical characterization of stabilized Ag nanospheres used in this study. All information provided by Nanotechnology Characterization Laboratory (NCL).

Nanosphere	Endotoxin (EU/mL)	Hydrodynamic Diameter by DLS (nm)	Core Diameter by TEM (nm)	Ag Concentration by ICP-MS (mg/g)	Zeta Potential (mV)	Medium Supplied In
20 nm citrate stabilized Ag	<0.03	24.0	20.3	1.1	−48	2 mM Citrate
20 nm PVP stabilized Ag	<2.2	26.0	20.5	1.1	−37	H_2_O
110 nm citrate stabilized Ag	<0.03	104.2	111.5	1.0	−43	2 mM Citrate
110 nm PVP stabilized Ag	<0.50	112.3	111.3	1.1	−26	H_2_O
